# Caregiver Stress and Social Determinants of Health: Comparing Alzheimer’s Disease Caregivers to Other Caregivers

**DOI:** 10.1093/geroni/igaf122.602

**Published:** 2025-12-31

**Authors:** Derek Falk, Elizabeth Vásquez, Anna Bokun, Christian Vazquez, Sunshine Rote

**Affiliations:** The University of Texas at Arlington, Arlington, Texas, United States; University at Albany (SUNY), Albany, New York, United States; The University of Texas at Austin, Boston, Massachusetts, United States; The University of Texas at Arlington, Arlington, Texas, United States; University of Louisville, Louisville, Kentucky, United States

## Abstract

Caregivers for individuals with Alzheimer’s Disease and related dementias (ADRD) experience excessive psychological and financial burdens compared to caregivers of other conditions due to the prolonged and unpredictable nature of dementia-related care. The impact of social determinants of health (SODH) on the levels and sources of stress experienced by caregivers of individuals with ADRD as compared to caregivers of individuals with other chronic conditions is unclear. Using the 2023 Behavioral Risk Factor Surveillance System (BRFSS), including the SDOH and health equity module, we assessed ADRD caregiver burden by sociodemographic and SODH measures. Univariable and bivariable statistics described the distribution of the caregivers and compared ADRD caregivers to caregivers of other chronic conditions. Weighted and adjusted multivariable logistic regressions assessed the odds of feeling stressed among caregivers. Over 25% of caregivers assisted with ADRD. Compared to caregivers of other chronic conditions, more ADRD caregivers were aged 55 + (P = 0.002) and female (P = 0.003). Our regression models showed food insecurity or not having food last and not being able to buy more when needed (odds ratio [OR]: 3.20; confidence interval [CI]: 2.29-4.48), financial strain or not being able to pay mortgage, rent, or utility bills (OR: 1.81; CI: 1.06-2.49), caregiving for someone with ADRD (OR: 1.40; CI: 1.06-1.85), and being female (OR: 1.55; CI: 1.28-1.87) were significantly associated with caregiver stress. Social determinants of health negatively impact caregiver stress. Our findings highlight the need for targeted interventions and supportive services that mitigate the psychological and financial burden faced by ADRD caregivers.

